# Biomechanical comparison of polyetheretherketone rods and titanium alloy rods in transforaminal lumbar interbody fusion: a finite element analysis

**DOI:** 10.1186/s12893-024-02462-8

**Published:** 2024-05-29

**Authors:** Jie Li, Shuai Cao, Bo Zhao

**Affiliations:** 1 Department of Orthopedics, Second Affiliated Hospital of Xi’an Jiaotong University, 157th West Fifth Road, Xi’an, 710004 Shaanxi Province China; 2 Department of Orthopedics, Civil Aviation General Hospital, No. 1, Gaojing Stress, Chaoyang District, Beijing, 100123 China

**Keywords:** Transforaminal lumbar interbody fusion, Finite element analysis, Polyetheretherketone rod, Titanium alloy rod

## Abstract

**Background:**

Whether polyetheretherketone (PEEK) rods have potential as an alternative to titanium alloy (Ti) rods in transforaminal lumbar interbody fusion (TLIF) remains unclear, especially in cases with insufficient anterior support due to the absence of a cage. The purpose of this study was to investigate biomechanical differences between PEEK rods and Ti rods in TLIF with and without a cage.

**Methods:**

An intact L1-L5 lumbar finite element model was constructed and validated. Accordingly, four TLIF models were developed: (1) Ti rods with a cage; (2) PEEK rods with a cage; (3) Ti rods without a cage; and (4) PEEK rods without a cage. The biomechanical properties were then compared among the four TLIF constructs.

**Results:**

With or without a cage, no obvious differences were found in the effect of PEEK rods and Ti rods on the range of motion, adjacent disc stress, and adjacent facet joint force. Compared to Ti rods, PEEK rods increase the average bone graft strain (270.8-6055.2 µE vs. 319.0-8751.6 µE). Moreover, PEEK rods reduced the stresses on the screw-rod system (23.1–96.0 MPa vs. 7.2–48.4 MPa) but increased the stresses on the cage (4.6–35.2 MPa vs. 5.6–40.9 MPa) and endplates (5.7–32.5 MPa vs. 6.6–37.6 MPa).

**Conclusions:**

Regardless of whether a cage was used for TLIF, PEEK rods theoretically have the potential to serve as an alternative to Ti rods because they may provide certain stability, increase the bone graft strain, and reduce the posterior instrumentation stress, which might promote bony fusion and decrease instrumentation failure.

**Supplementary Information:**

The online version contains supplementary material available at 10.1186/s12893-024-02462-8.

## Background

Since its initial introduction in 1982, transforaminal lumbar interbody fusion (TLIF) has been one of the most commonly used lumbar fusion procedures for the treatment of lumbar degenerative diseases [1–3]. During surgical procedures, the titanium alloy (Ti) pedicle screw-rod fixation system has been used to ensure segmental stability immediately after surgery and to promote solid fusion [4, 5]. However, the high elastic modulus of Ti (110 GPa) in the fixation system excessively increases the stiffness of the posterior column, which has been considered an important factor causing adjacent segment degeneration (ASD) [6, 7].

To address this issue, polyetheretherketone (PEEK) rods have been introduced [8]. The elastic modulus of PEEK was reported to be only 3.6 GPa, which is much lower than that of Ti [9]. Thus, as flexible materials, PEEK rods may significantly reduce the stiffness of the fixation system and mimic the physiological load distribution of the normal spine [10, 11]. Theoretically, a semi-rigid PEEK rod system increases load sharing on the anterior column, which helps to facilitate fusion rates according to Wolff’s law [7, 12]. It also reduces stress concentration on screw-rod systems and decreases adjacent structure pressure compared with rigid Ti rods [13]. Previous studies have investigated the PEEK rods in anterior, posterior lumbar interbody fusion, and posterolateral fusion [14–17]. However, few published studies have evaluated the biomechanical differences between PEEK rods and Ti rods in TLIF.

In addition, cages filled with bone grafts are widely used for interbody fusion to restore disc height and improve fusion rates because of their superior mechanical strength [18, 19]. However, when the endplate is damaged, the placement of a cage may increase the risks of endplate collapse and cage subsidence. In such cases, the use of large bone blocks for interbody fusion is a good choice because the elastic modulus of the bone block is lower than that of the cage, which may reduce the above complications [20–22]. Furthermore, the use of bone blocks can reduce hospitalization costs [23, 24]. Nevertheless, whether PEEK rods function well and have the potential to serve as an alternative to Ti rods in the absence of a cage remains unreported. Given the variability in the use of cages in TLIF, a thorough biomechanical investigation of these two different rods is necessary.

To this end, four TLIF models were constructed using a finite element (FE) method (Ti rods with a cage, PEEK rods with a cage, Ti rods without a cage, and PEEK rods without a cage). Then, the segmental ROMs, instrumentation stresses, endplate and disc stresses, bone graft strain, and facet joint force (FJF) among the four configurations were analyzed and compared. This study provides a comprehensive description of the biomechanical properties of PEEK rods and Ti rods in TLIF with and without a cage, and the results may provide a theoretical background for the application of PEEK rods.

## Methods

### FE modeling of the intact L1-L5 lumbar spine

L1-L5 vertebrae computed tomography (CT) data were obtained from a healthy 29-year-old male subject (height: 176 cm, weight: 60 kg) with no history of spine-related disease or trauma. This study was approved by the ethics committee of our hospital, and informed consent was obtained from the subject. The procedure for lumbar model reconstruction was similar to that applied in previous studies [25]. Thin-layer (0.625 mm) CT data were saved in DICOM format and imported into Mimics (Materialise, Inc., Leuven, Belgium) to generate a surface model. The solid model was constructed using Materialise 3-Matic software (Materialise, Inc., Leuven, Belgium). The mesh models and ligamentous structures were created using HyperMesh (Altair Engineering, Inc., Troy, Michigan, USA). The conditions for the material properties, model assembly, and analysis were defined using Abaqus (Hibbitt, Karlsson, and Sorensen, Inc., Providence, Rhode Island, USA). The material properties and element types used in the FE models were defined based on previous studies and shown in Table 1 [26, 27].

**Table 1:** Definition of materials properties in the finite element models

Materials	Element type	Young’s modulus (MPa)	Poisson’s ratio (µ)	Cross-Sectional Area (mm2)
Bone				
Cortical bone	C3D4	12,000	0.3	
Cancellous bone	C3D4	100	0.2	
Bony endplate	C3D8I	1 200	0.29	
Cartilage endplate	C3D8I	24	0.4	
Intervertebral disc				
Nucleus pulposus	C3D8I	1	0.49	
Annulus ground	C3D8H	Hyperelastic C10 = 0.18 C01 = 0.045		
Annulus fiber	T3D2	Hypoelastic (360–550 MPa)		
Ligaments	T3D2	Hypoelastic		
Anterior longitudinal		7.8 (< 12%), 20 (> 12%)		63.7
Posterior longitudinal		10 (< 11%), 20 (> 11%)		20
Ligamentum flavum		15 (< 6.2%), 19.5 (> 6.2%)		40
Supraspinous		8.0 (< 20%), 15 (> 20%)		30
Interspinous		10 (< 14%), 11.6 (> 14%)		40
Intertransverse		10 (< 18%), 58.7 (> 18%)		1.8
Capsular		7.5 (< 25%), 32.9 (> 25%)		66
Screws	C3D4	110,000	0.3	
Rods				
Ti-6Al-4 V	C3D4	110,000	0.3	
PEEK	C3D4	3600	0.25	
Cage	C3D4	3600	0.25	
Bone grafts	C3D4	100	0.2	

Ti: Titanium; Al: Aluminum; V: Vanadium; PEEK: polyetheretherketone; C3D4: 4-node tetrahedral elements; C3D8: 8-node hexahedral elements; T3D2: 2-node truss elements

**Fig. 1:**
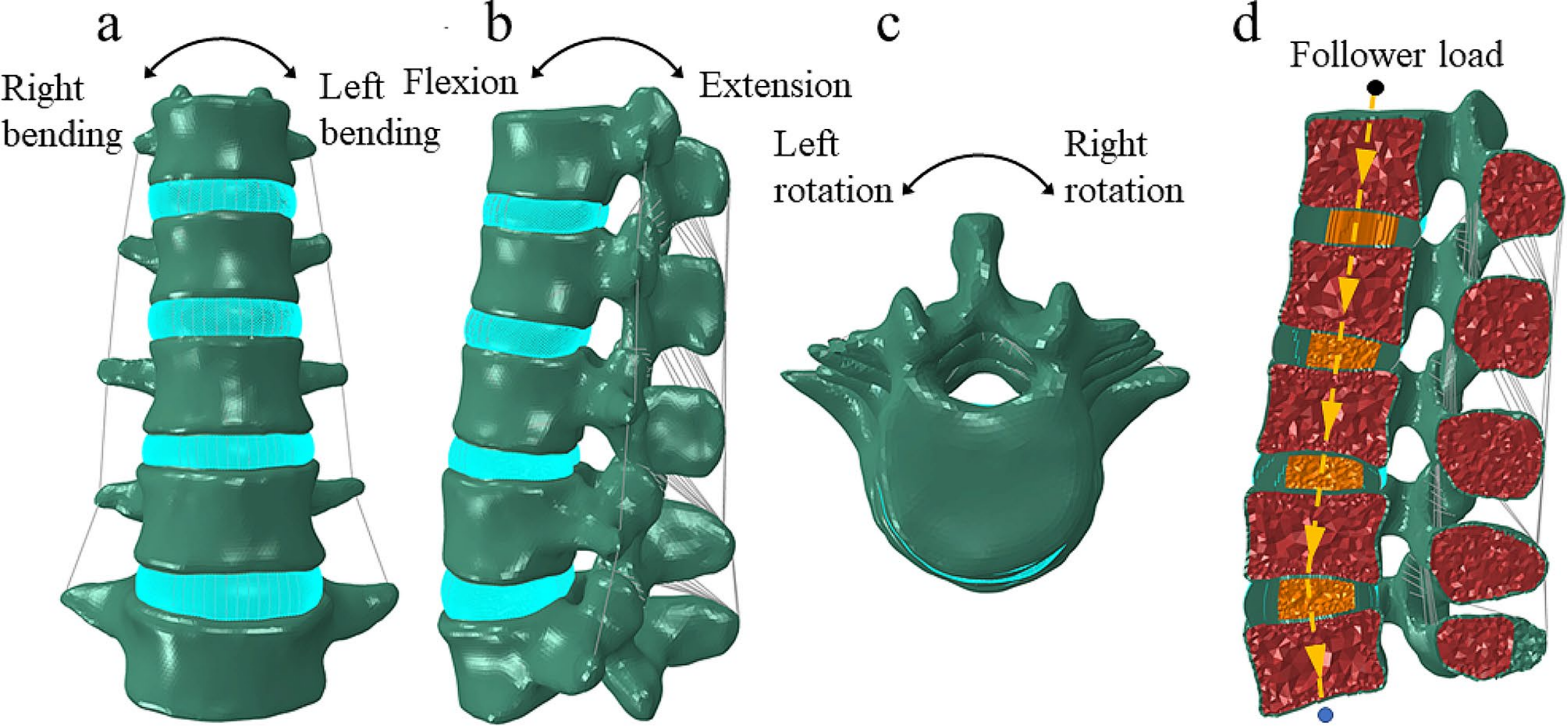
The intact L1-L5 finite element model of the human spine. (**a**) front view, (**b**) lateral view, (**c**) top view, and (**d**) longitudinal section. The yellow dashed line is the follower load path

As in the case in a previous study including a lumbar FE model, 1-mm-thick cortical shells and bony endplates covered the surfaces of the vertebral body (Fig. 1) [28]. The thickness of the cartilage endplates was 0.5 mm. The nucleus pulposus was simulated as a homogeneous elastic element, which accounted for 40% of the intervertebral disc volume [29]. The annulus fibrosus was constructed using a heterogeneous fiber-reinforced composite consisting of annulus fibers and a ground substance [30]. The ligamentous structures included the anterior longitudinal, posterior longitudinal, flavum, supraspinous, interspinous, intertransverse, and capsular ligaments [31]. They were modeled as tension-only truss elements and were simulated as hypoelasticity materials.

**Fig. 2:**
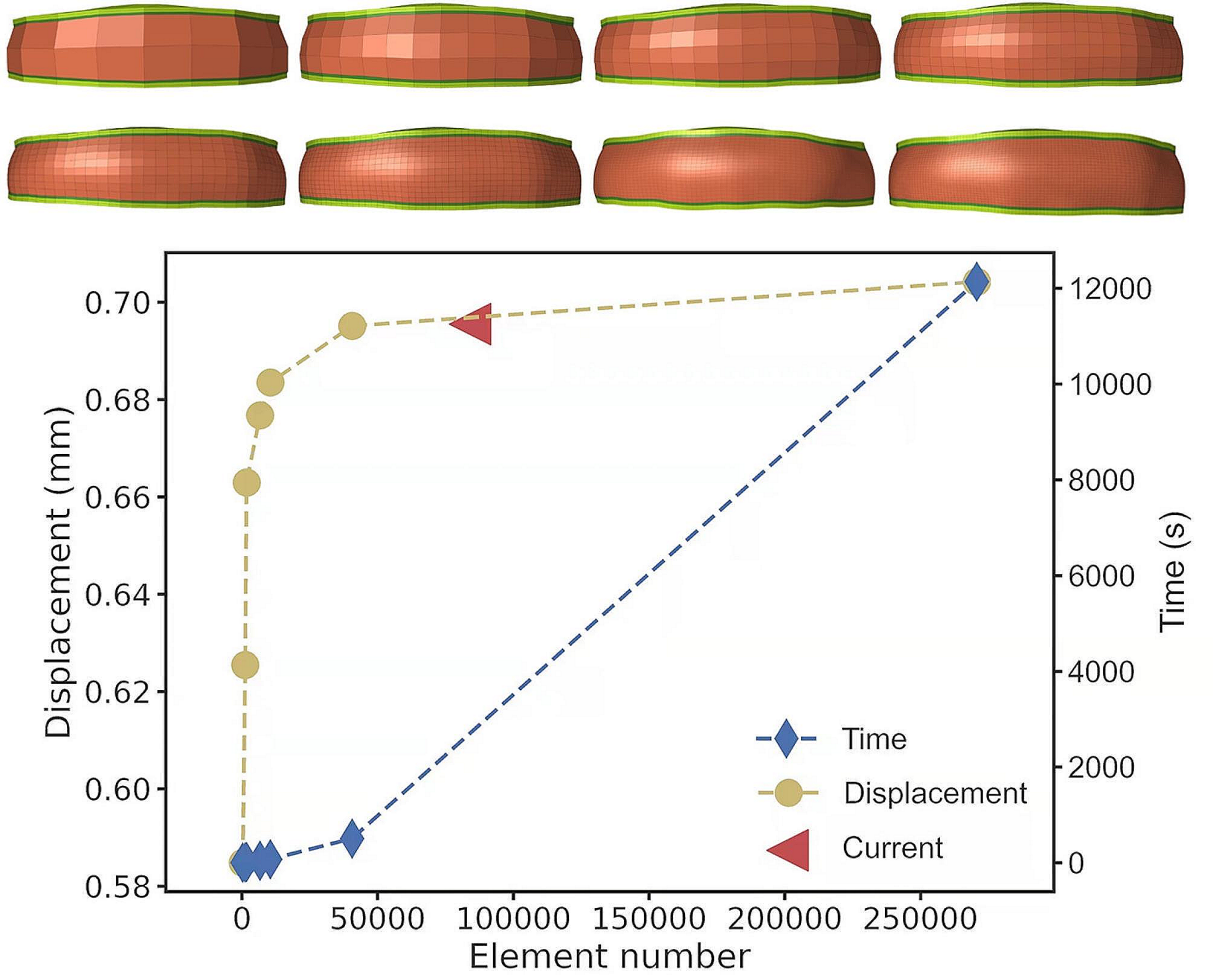
Mesh convergence test was performed by repeating the solution with eight different element size meshes. The blue dotted line represents the relationship between the computation time and element number, the yellow dotted line represents the relationship between simulation results and element number, and the red triangle represents the chosen mesh size (84,002 elements) in the current study

Convergence analysis was performed on a part of the intact model, including the fibrous annulus, nucleus pulposus, cartilage endplate, and cortical bone. Generally, the accuracy of the solution increased as the mesh size decreased but at the expense of computation time. To achieve a compromise between high accuracy and a short computation time, the current mesh size of the model was chosen (Fig. 2). Additional mesh refinement hardly affected the results but increased the time cost dramatically.

### FE modeling of the TLIF constructs

**Fig. 3:**
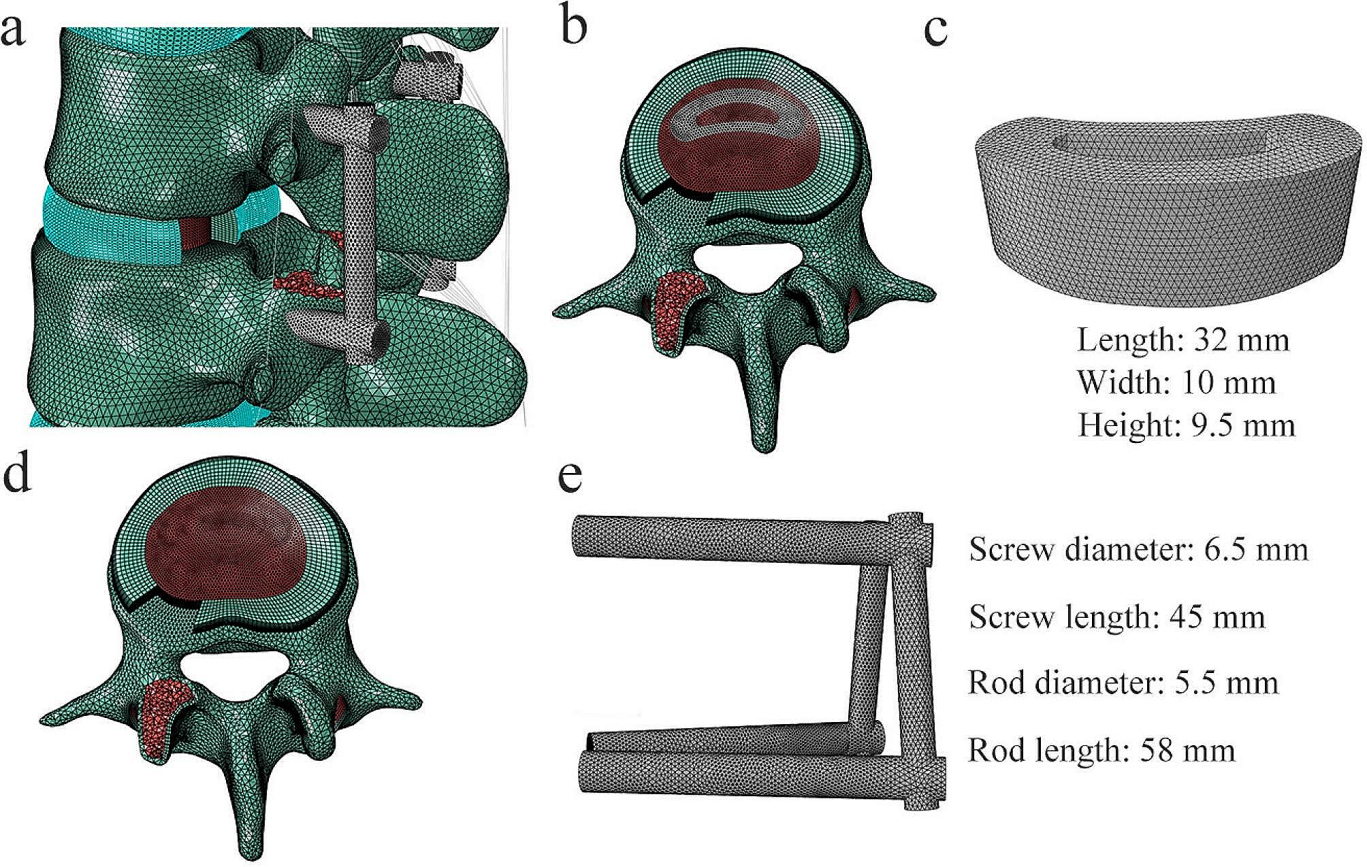
The surgical model for transforaminal lumbar interbody fusion. (**a**) left posterior oblique view, (**b**) model with a cage, (**c**) cage, (**d**) model without a cage, and (**e**) pedicle screw–rod fixation

In this study, four TLIF constructs were constructed based on the intact model: (1) Ti rods with a cage; (2) PEEK rods with a cage; (3) Ti rods without a cage; and (4) PEEK rods without a cage. To simulate the processes of decompression and fusion, a left L3/4 facetectomy was performed; then, the entire nucleus pulposus, the left posterior part of the annulus fibrosus, and capsular and flavum ligaments were removed from all surgical models (Fig. 3a). A banana-shaped PEEK cage (length, 32 mm; width, 10 mm; height, 9.5 mm) was installed on the anterior part of the L3/4 intervertebral space (Fig. 3b-c) [31]. Cancellous bone was implanted into the inner and outer spaces of the cage to fill the intervertebral space. To eliminate the overlap between the cage and endplates, a Boolean operation was performed. In the models without cages, cancellous bone was used to fill in the L3/4 space (Fig. 3d). Regarding posterior fixation, the pedicle screw-based fixation system consisted of four screws (diameter, 6.5 mm; length, 45 mm) and two connecting rods (diameter, 5.5 mm; length, 58 mm) (Fig. 3e) [32]. The interfaces of the cage-endplate, cage-graft, graft-endplate, bone-screw, and screw-rod were set as a fully bonded condition via node sharing [27].

### Loading conditions

The inferior surface of the L5 vertebra for all models was constrained. A 400 N follower load was applied to mimic the in vivo muscle forces and the weight of the upper torso of a normal adult. The method of applying the follower load referred to the published research by Sun et al. [33]. Additional 8-Nm bending moments were applied to the L1 vertebra to simulate motion in the coronal, sagittal, and axial planes (extension, flexion, left lateral bending, and left axial rotation). Furthermore, the ROMs of each segment were compared with previously reported values to validate the intact model [25, 34–36]. Finally, the ROMs at the L2/3, L3/4, and L4/5 levels, maximum disc stresses and FJF at the L2/3 and L4/5 levels, average bone graft strain, and maximum stresses on the L3/4 endplates, cage, screws, rods, and bone-screw interfaces among the four configurations were analyzed. The left and right facet joint forces were averaged during extension and flexion. The forces from loaded facets were recorded during lateral bending and axial rotation.

## Results

### Model validation

**Fig. 4:**
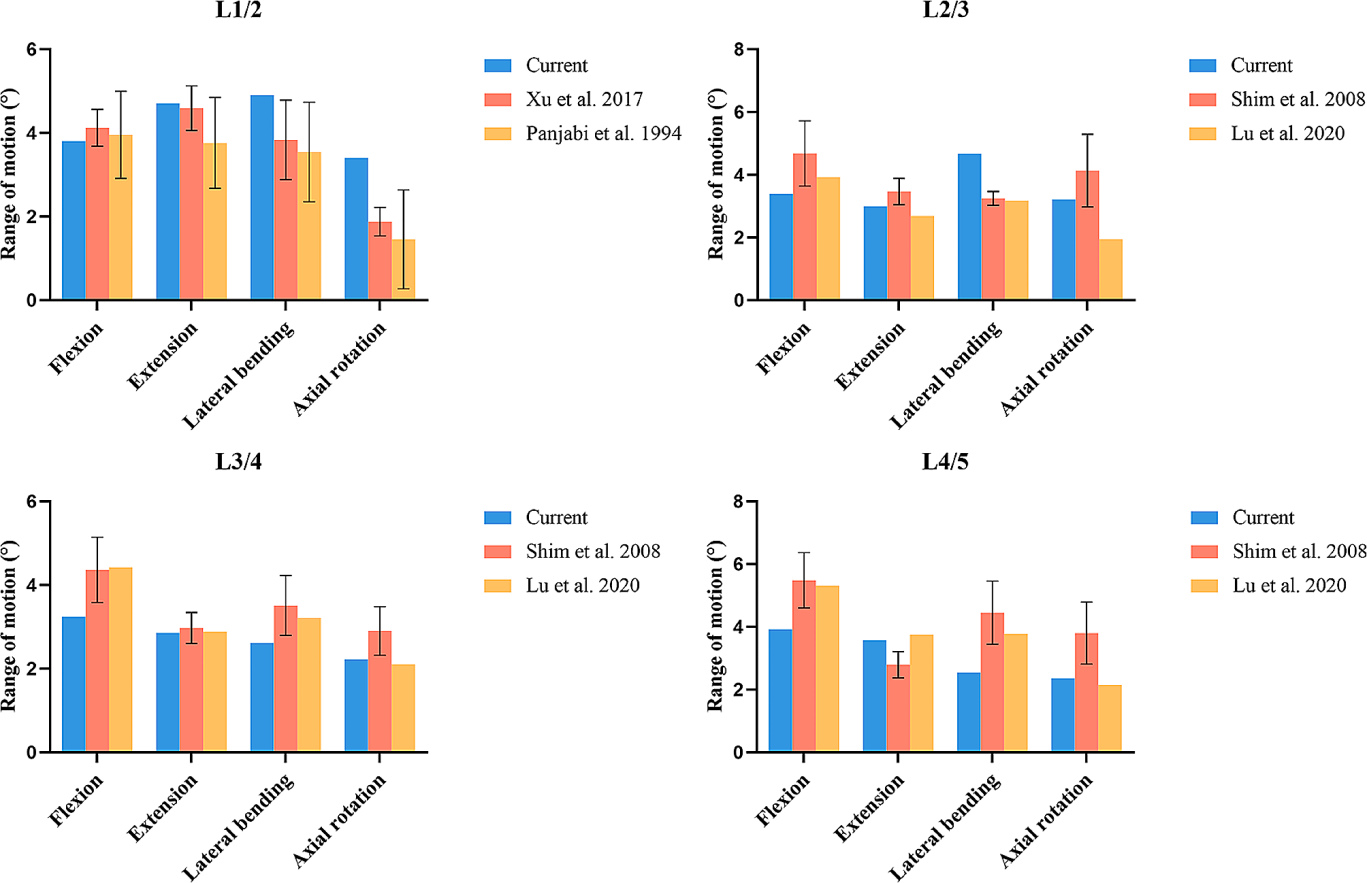
Comparison of range of motion between the current intact model and the previous studies

Under extension, flexion, lateral bending, and axial rotation, the results of ROMs were compared with the previous experimental and FE results, as shown in Fig. 4 [25, 34–36]. The agreement between the ROMs obtained under similar loading conditions in this study and previous studies, including cadaver and FE studies, was acceptable, indicating that the proposed model was suitable for application in future studies.

### ROM

**Fig. 5:**
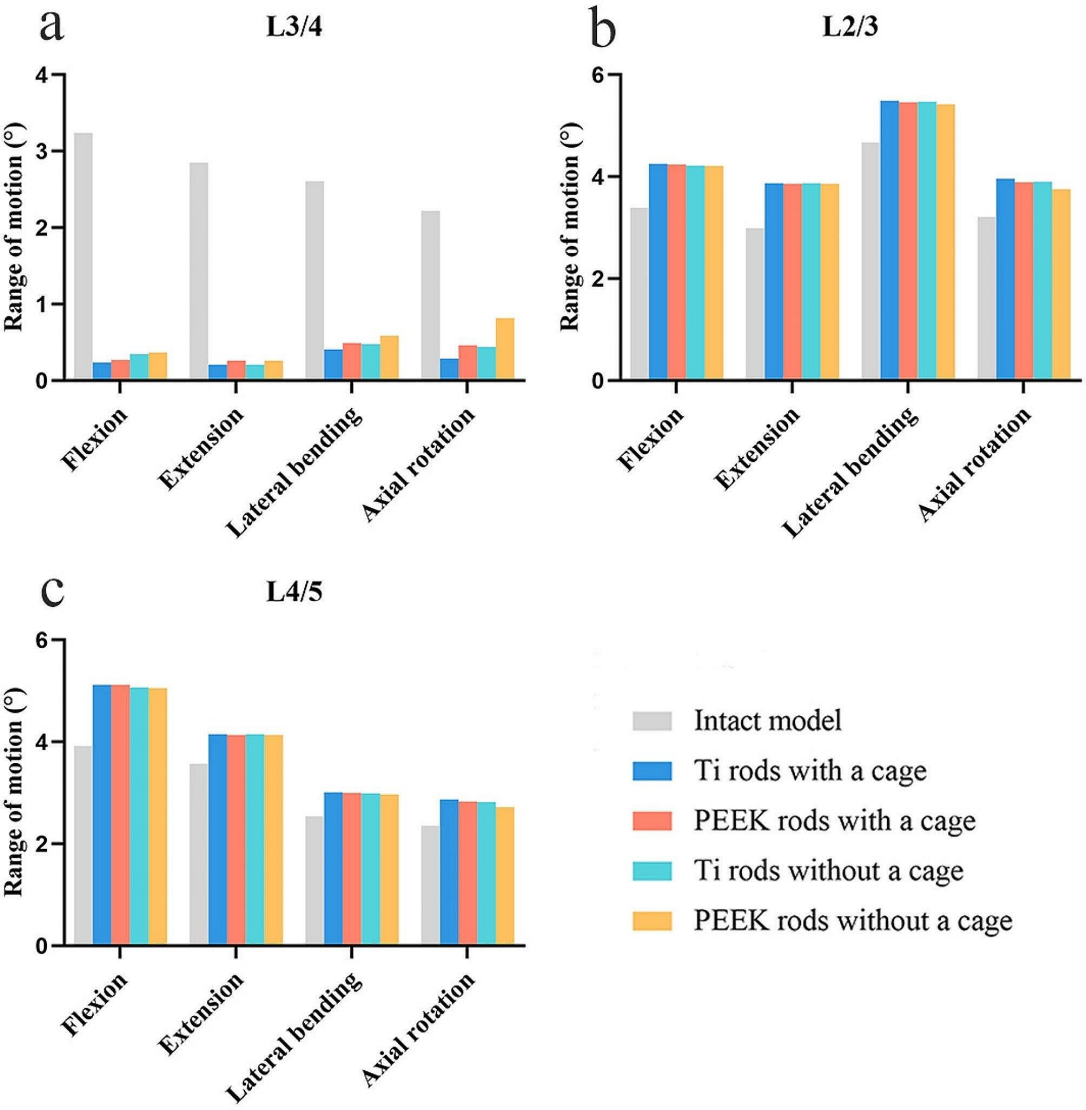
Range of motion in four surgical models at (**a**) L3/4 segment, (**b**) L2/3 segment, and (**c**) L4/5 segment

For all surgical models, the predicted ROMs in all motions substantially decreased at the surgical segment and increased at adjacent segments compared with the intact model. In the intact model and surgical models (Ti rods with a cage, PEEK rods with a cage, Ti rods without a cage, and PEEK rods without a cage), the ROM values were 2.22°-3.24°, 0.21°-0.41°, 0.26°-0.49°, 0.21°-0.48°, and 0.26°-0.82° at the L3/4 segment (Fig. 5a), respectively; 2.99°-4.67°, 3.87°-5.49°, 3.86°-5.46°, 3.87°-5.47°, and 3.76°-5.42° at the L2/3 segment (Fig. 5b), respectively; and 2.36°-3.92°, 2.87°-5.12°, 2.83°-5.12°, 2.82°-5.07°, and 2.72°-5.06° at the L4/5 segment (Fig. 5c), respectively. Generally, little difference in the ROMs at surgical and adjacent segments was noted among the four surgical models.

### The average strain of the bone grafts

**Fig. 6:**
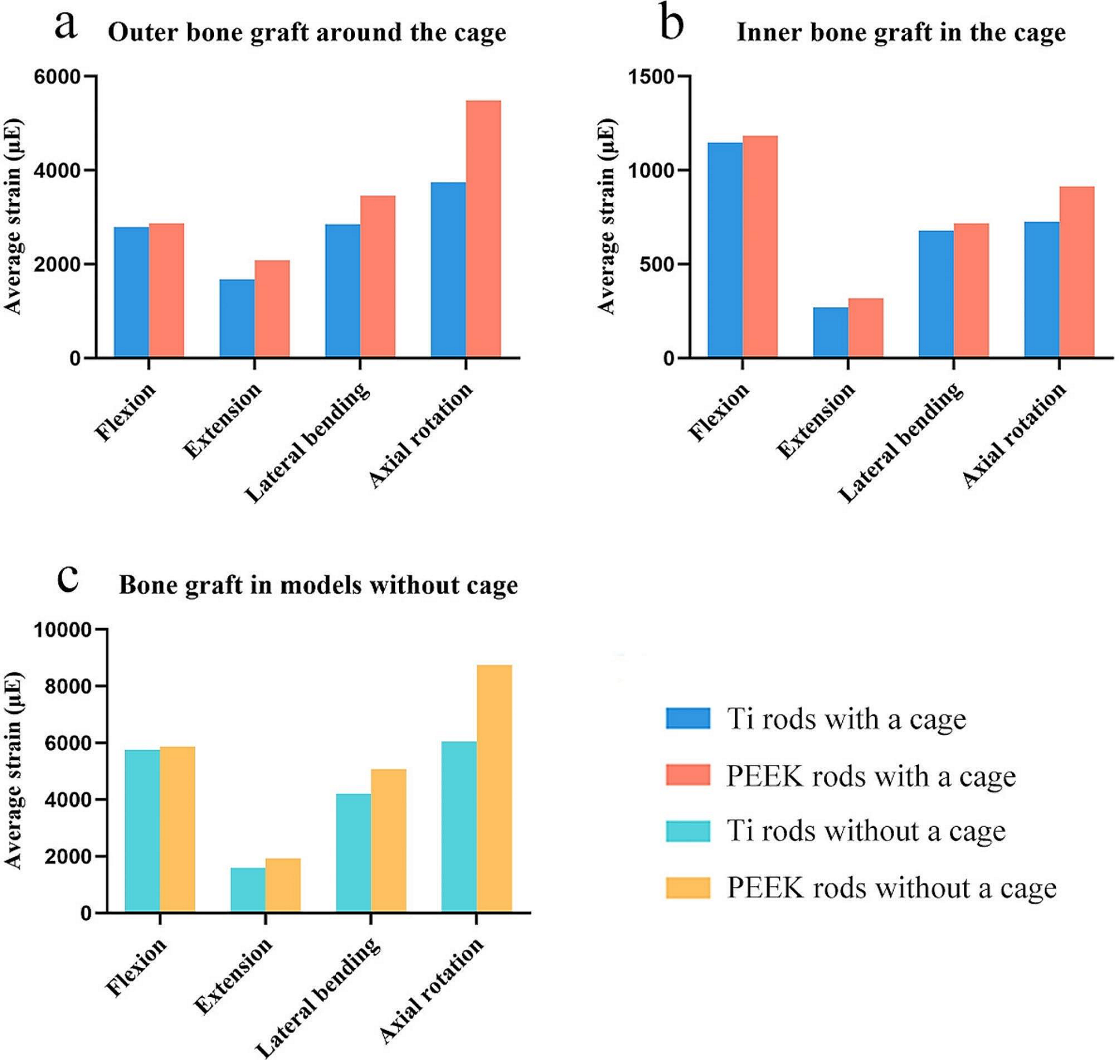
Average strain of the (**a**) outer bone grafts around the cage, (**b**) inner bone grafts in the cage, and (**c**) bone grafts in models without a cage

In the models featuring Ti rods with a cage and PEEK rods with a cage, the average strains were 1676.7-3747.8 µE and 2089.8-5491.3 µE for the outer bone grafts around the cage, respectively (Fig. 6a), and 270.8-1148.4 µE and 319.0-1184.3 µE for the inner bone grafts in the cage, respectively (Fig. 6b). In the models featuring Ti rods without a cage and PEEK rods without a cage, the average strains of the bone grafts were 1592.8-6055.2 µE and 1930.1-8751.6 µE, respectively (Fig. 6c). The nephograms of von Mises stress on the bone grafts are shown in Figure S1.

### Disc stress and FJF at adjacent segments

**Fig. 7:**
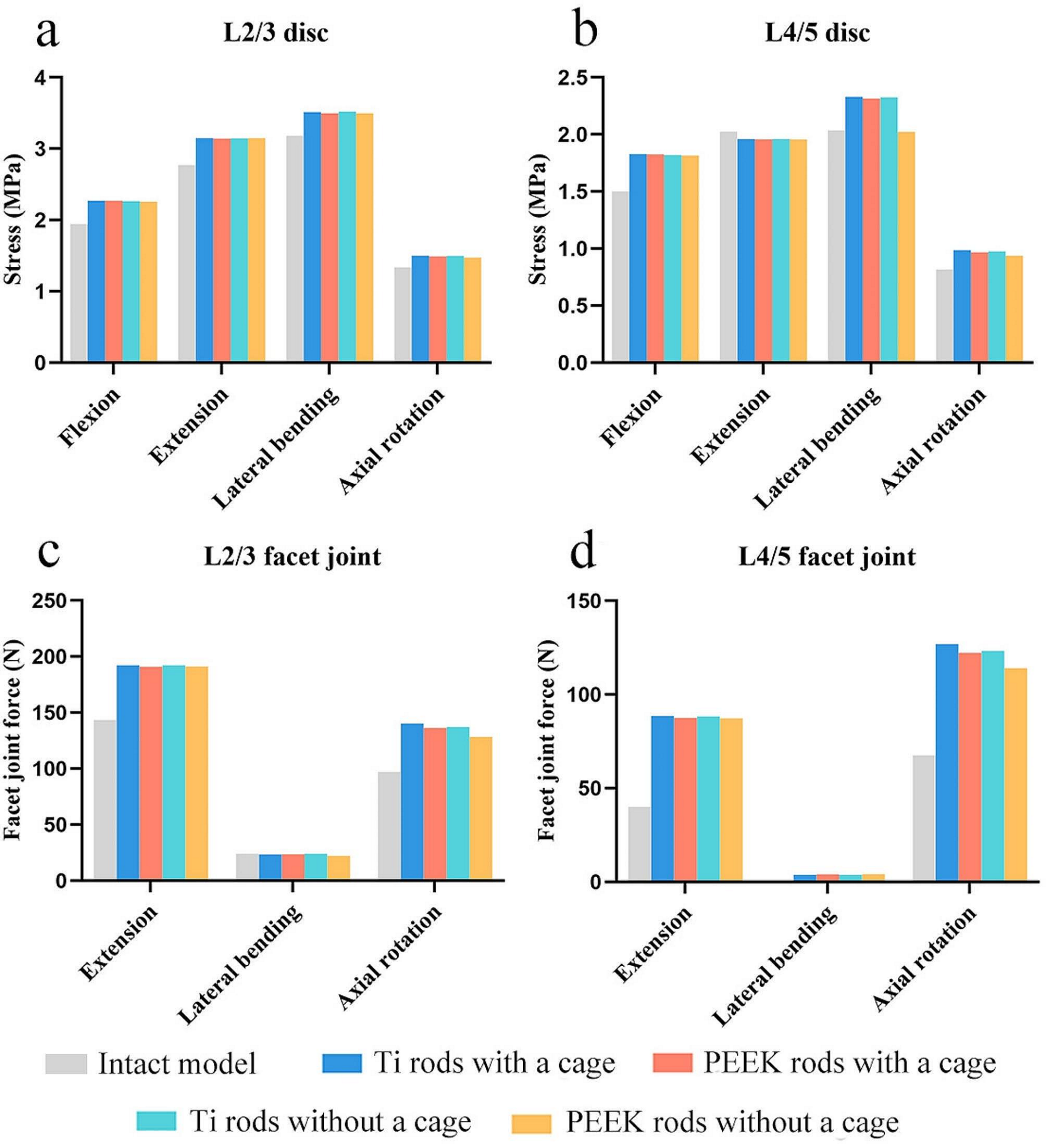
Maximum von Mises stresses of the (**a**) L2/3 disc and (**b**) L4/5 disc, and maximum force of the (**c**) L2/3 facet joints and (**d**) L4/5 facet joints in the intact model and surgical models

For all surgical models, the maximum disc stresses and FJF at the L2/3 and L4/5 segments were found to be higher than those obtained for the intact model. The maximum disc stresses among the four surgical models were almost equal (1.5–3.5 MPa at the L2/3 segment and 0.9–2.3 MPa at the L4/5 segment) (Fig. 7a-b). The FJF results were also very similar (22.2–192.2 N at the L2/3 segment and 3.8–127.0 MPa at the L4/5 segment) (Fig. 7c-d). The nephograms of von Mises stress on the L2/3 and L4/5 discs are shown in Figure S2-S3.

### Cage and endplate stress

**Fig. 8:**
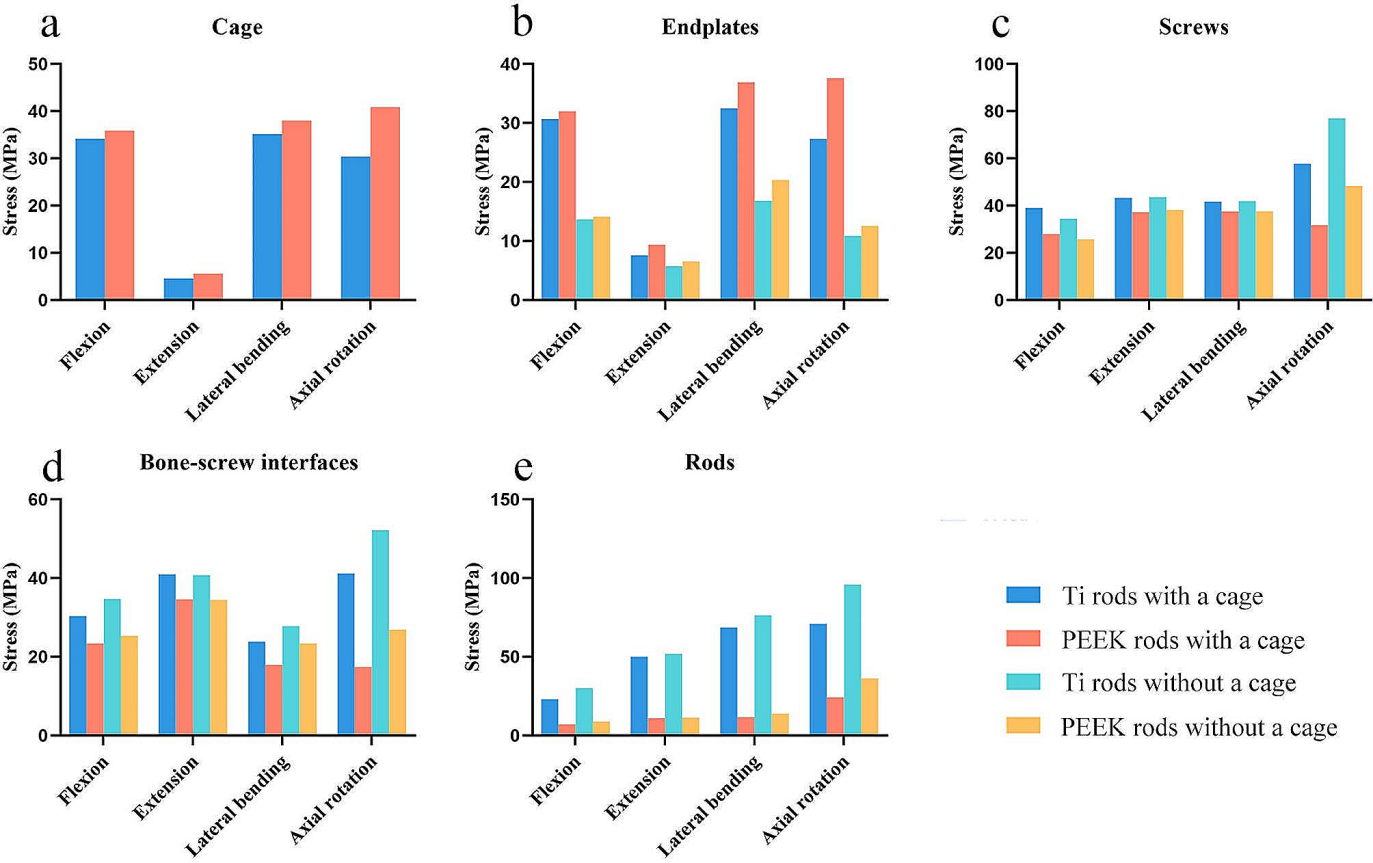
Maximum von Mises stresses of the (**a**) cage, (**b**) endplates, (**c**) screws, (**d**) bone-screw interfaces, and (**e**) rods in the four surgical models

The maximum stresses on the cage in the models featuring Ti rods with a cage and PEEK rods with a cage ranged from 4.6 MPa to 35.2 MPa and 5.6 MPa to 40.9 MPa, respectively (Fig. 8a). The maximum stresses applied to the L3/4 endplates were 7.6–32.5 MPa and 9.4–37.6 MPa for the models featuring Ti rods with a cage and PEEK rods with a cage, respectively, which were much larger than those for the models featuring Ti rods without a cage (5.7–16.9 MPa) and PEEK rods without a cage (6.6–20.3 MPa) (Fig. 8b), especially in flexion, lateral bending, and axial rotation. The nephograms of von Mises stress on the cage and endplate are shown in Figure S4-S5.

### Posterior instrumentation stress

In the models featuring Ti rods with a cage, PEEK rods with a cage, Ti rods without a cage, and PEEK rods without a cage, the largest stresses ranged from 39.1 to 57.8 MPa, 27.9 to 37.6 MPa, 34.5 to 77.1 MPa, and 25.7 to 48.4 MPa for screws, respectively (Fig. 8c), and 23.9 to 41.2 MPa, 17.4 to 34.6 MPa, 27.8 to 52.2 MPa, and 23.4 to 34.5 MPa for bone-screw interfaces, respectively (Fig. 8d). The maximum stresses experienced by the rods were 7.2–24.3 MPa and 9.0-36.3 MPa in the models featuring PEEK rods with a cage and PEEK rods without a cage, respectively, which were much lower than those in the models featuring Ti rods with a cage (23.1–70.9 MPa) and Ti rods without a cage (30.1–96.0 MPa) (Fig. 8e). The ratio of peak stress to yield stress (Ti, 750 MPa; PEEK, 100 MPa) for the PEEK rods (7.2-36.3%) was higher than that for the Ti rods (3.1-12.8%). The nephograms of von Mises stress on the posterior instrumentation are shown in Figure S6.

## Discussion

As reported, PEEK rods with biocompatible and radiolucent characteristics may reduce the stiffness of the pedicle screw-rod system compared to Ti rods [7, 10, 12]. However, studies comparing PEEK rods and Ti rods with respect to biomechanics in TLIF are lacking, especially for cases of insufficient anterior support due to the absence of a cage (bone grafts alone). In our study, we found that PEEK rods have the potential to serve as an alternative to Ti rods in TLIF from a biomechanical perspective regardless of cage use.

One of the major objectives of TLIF is to provide postoperative stability of the spine through the implantation of an interbody cage and posterior screw-rod system. We found that although Ti rods had an advantage over PEEK rods in restricting segmental mobility, these differences were negligible. TLIF models with a cage decreased the ROMs of the fixed segment by 79.3–92.6% under all loading conditions compared with the intact model, suggesting that the ability of PEEK rods to stabilize the spine is as good as that of Ti rods when a cage is present. This result is similar to those in previous reports by Hsieh et al. [9]. One reason may be the favorable biomechanical environment and spinal stability provided by the cage. Another possible reason for this finding was that the difference in rigidity between the PEEK and Ti rods had only a minor effect on the segmental ROM unless the construct stiffness was very low [8]. The results of a cadaveric biomechanical test conducted by Gornet et al. also showed no significant difference in the stability provided by PEEK and Ti rods after partial discectomy and hemi-facetectomy [37]. In the TLIF models without a cage, similar results were obtained, demonstrating that the simultaneous use of PEEK rods and bone grafts in TLIF may provide certain stability, especially in flexion and extension.

According to the mechanostat hypothesis proposed by Frost, a certain degree of strain may excite a positive adaptive response (bone modeling) to mechanical overloading, and strains below the no-response threshold will cause a negative adaptive response (bone remodeling) [38]. In the models with and without a cage, PEEK rods increased the average strain of the bone grafts relative to Ti rods, especially during axial rotation. The excellent load-sharing characteristics of PEEK rods may weaken the stress-shielding effect on interbody bone grafts to promote interbody fusion or reduce the risk of pseudarthrosis. Wang et al. also found that PEEK rods achieved better fusion than Ti rods after posterior bone graft fusion and internal fixation in canines [39]. In addition, our study showed that the models without a cage generated a greater average strain of the bone grafts compared to the models with a cage. The reason was that the cage increased the stress-shielding effect on the interbody bone grafts because the elastic modulus of the PEEK cage (3600 MPa) was much larger than that of bone grafts (100 MPa) [40]. Consistently, Lin et al. revealed that the fusion rate among patients treated with a PEEK cage was slightly lower than that of patients treated with autologous bone at 8–12 weeks postoperatively (94.1% vs. 97.1%) [41]. We also demonstrated that the average strain of the bone grafts of the four TLIF constructs was considerably smaller than the fracture strain value for bone (25,000 µE), but the optimal strain for bone growth requires further exploration [42].

The use of a cage for interbody fusion is conducive to restoring disc space height, maintaining spinal stability, and enhancing the load-bearing capacity of the anterior column [18]. However, cage failure and endplate collapse are common postoperative complications related to the cage. Regrettably, PEEK rods induced larger stresses on the cage and endplates than Ti rods because they transferred more load to the anterior column of the spine, which might increase the risks of cage failure and endplate collapse. Nevertheless, we revealed that the models without a cage generated much lower endplate stress under all load conditions compared to the models with a cage, regardless of the rod materials. The reason can be attributed to the small contact area between the cage and endplates, which aggravates the concentration of stress on the endplates. From this perspective, a therapeutic strategy combining PEEK rods and bone grafts might be a feasible option. However, whether PEEK rods and bone grafts with insufficient mechanical strength can provide adequate anterior structural support and maintain the disc height remains unclear because of the lack of research in this area.

Some researchers believe that low back pain is caused by abnormal load transfer rather than abnormal ROM [16]. PEEK rods have superior performance in balancing the load distribution between the anterior and posterior columns of the spine. In the models with and without a cage, PEEK rods decreased the maximum stresses applied to the screw, rod, and bone–screw interfaces compared to Ti rods, especially during axial rotation, which was possible to reduce the risk of screw breakage and loosening for patients with osteoporosis [43]. Moreover, the maximum amounts of stress applied to the screws (48 MPa) and bone–screw interfaces (35 MPa) in the models fixed with PEEK rods were significantly lower than the strengths of the corresponding screws (750 MPa) and cortical bone (80–150 MPa) [44]. Gornet et al. demonstrated that PEEK rod loads were at least 6% less than Ti rod loads under all loading conditions [37]. Fan et al. showed that although PEEK rods reduced the stress on the rods compared to Ti rods, the ratio of peak stress to yield stress for the PEEK rods was higher [14]. The ratios obtained in this study were within the ranges of 7.2–36.3% for PEEK rods and 3.1–12.8% for Ti rods, demonstrating that PEEK rods may have a higher fracture risk. To date, however, no reports on PEEK rod fracture events are available in the literature.

ASD is a common long-term complication of lumbar fusion. Abnormal motion (quality and quantity), intervertebral disc pressure (IDP), and FJF of adjacent segments are closely related to ASD [45]. Wangsawatwong et al. showed that the use of pedicle screw–rod fixation can significantly affect the mobility of the adjacent segments [46]. Cunningham et al. found that spinal instrumentation increased the proximal IDP by as much as 45% during in vitro biomechanical tests [47]. Similarly, we found that the ROMs, disc stresses, and FJF at the adjacent segments were significantly higher in all surgical models than in the intact model, indicating that ASD was an inevitable process after spinal fusion and fixation. Jin et al. demonstrated that although both PEEK and Ti rods increased the intersegmental rotation and IDP in the upper adjacent segments, PEEK rods induced smaller-scale changes than Ti rods [48]. Athanasakopoulos et al. reported a retrospective clinical study of 52 patients who had posterior lumbar internal fixation systems with PEEK rods, where no ASD was observed after a mean follow-up period of 3 years [49]. Although the ROMs, disc stresses, and FJF at both the cephalad and caudal adjacent levels in the models with PEEK rods were better than those in the models with Ti rods, the differences were very small among the four surgical models in our study. Further high-quality studies are warranted to validate the effect of PEEK rods on ASD.

This study has several limitations. First, the screws were simplified into cylinders without thread, and the interfaces of the cage-endplate, cage-graft, graft-endplate, bone-screw, and screw-rod were set as a fully bonded condition. Thus, the FE model could not truthfully reflect the in vivo situation. Second, model building was based on published data. And there were some differences in the loading conditions between the current study and the literature. Therefore, the validation of the intact model was not sufficient. Third, our data were obtained from a healthy, young male subject. However, the human lumbar spine of each individual is unique and dependent on age, the presence of disease, and other factors. Finally, different loading protocols may influence the simulation results in FE analysis. Both the load-controlled and the displacement-controlled methods should be considered to obtain more realistic results [50].

## Conclusion

With or without a cage, we found no obvious differences in the effect of PEEK rods and Ti rods on ROMs, adjacent disc stress, and adjacent FJF. PEEK rods might promote interbody fusion by increasing the average bone graft strain. PEEK rods reduced the stresses on the screw-rod system but increased the ratio of peak stress to yield stress for rods and stresses on the cage and endplates, implying that they might decrease screw failure but increase the risks of rod fracture, cage damage, and endplate collapse. Overall, regardless of whether a cage is used for TLIF, PEEK rods have the potential to serve as an alternative to Ti rods, and more studies are needed to verify these results.

### Electronic supplementary material

Below is the link to the electronic supplementary material.

Supplementary Material 1: The nephograms of von Mises stress on the bone grafts

Supplementary Material 2: The nephograms of von Mises stress on the L2/3 disc

Supplementary Material 3: The nephograms of von Mises stress on the L4/5 disc

Supplementary Material 4: The nephograms of von Mises stress on the cage

Supplementary Material 5: The nephograms of von Mises stress on the endplates

Supplementary Material 6: The nephograms of von Mises stress on the posterior instrumentation

## Data Availability

The datasets used during the current study are available from the corresponding author on reasonable request.

## Abbreviations

FE: Finite element
PEEK: Polyetheretherketone
Ti: Titanium alloy
CT: Computed tomography
ROM: Range of motion
IDP: Intervertebral disc pressure
FJF: Facet joint force
ASD: Adjacent segment degeneration
TLIF: Transforaminal lumbar interbody fusion
