# Supplementary figures and images for: Biomechanical comparison of polyetheretherketone rods and titanium alloy rods in transforaminal lumbar interbody fusion: a finite element analysis
Source: BMC Surg. 2024 May 29;24:169. doi: 10.1186/s12893-024-02462-8 (PMC11134660; doi:10.1186/s12893-024-02462-8)

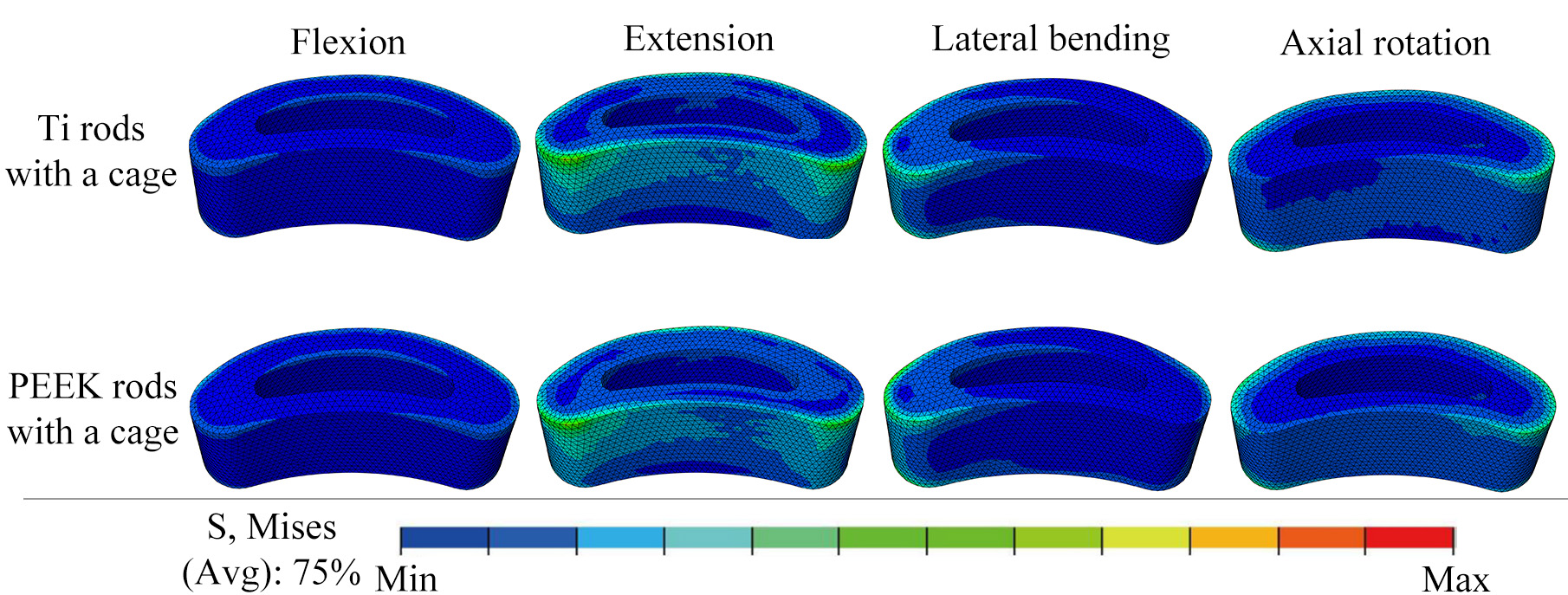

Supplement: Supplementary file 1 — Supplementary Material 1: The nephograms of von Mises stress on the bone grafts [file 12893_2024_2462_MOESM1_ESM.jpg]

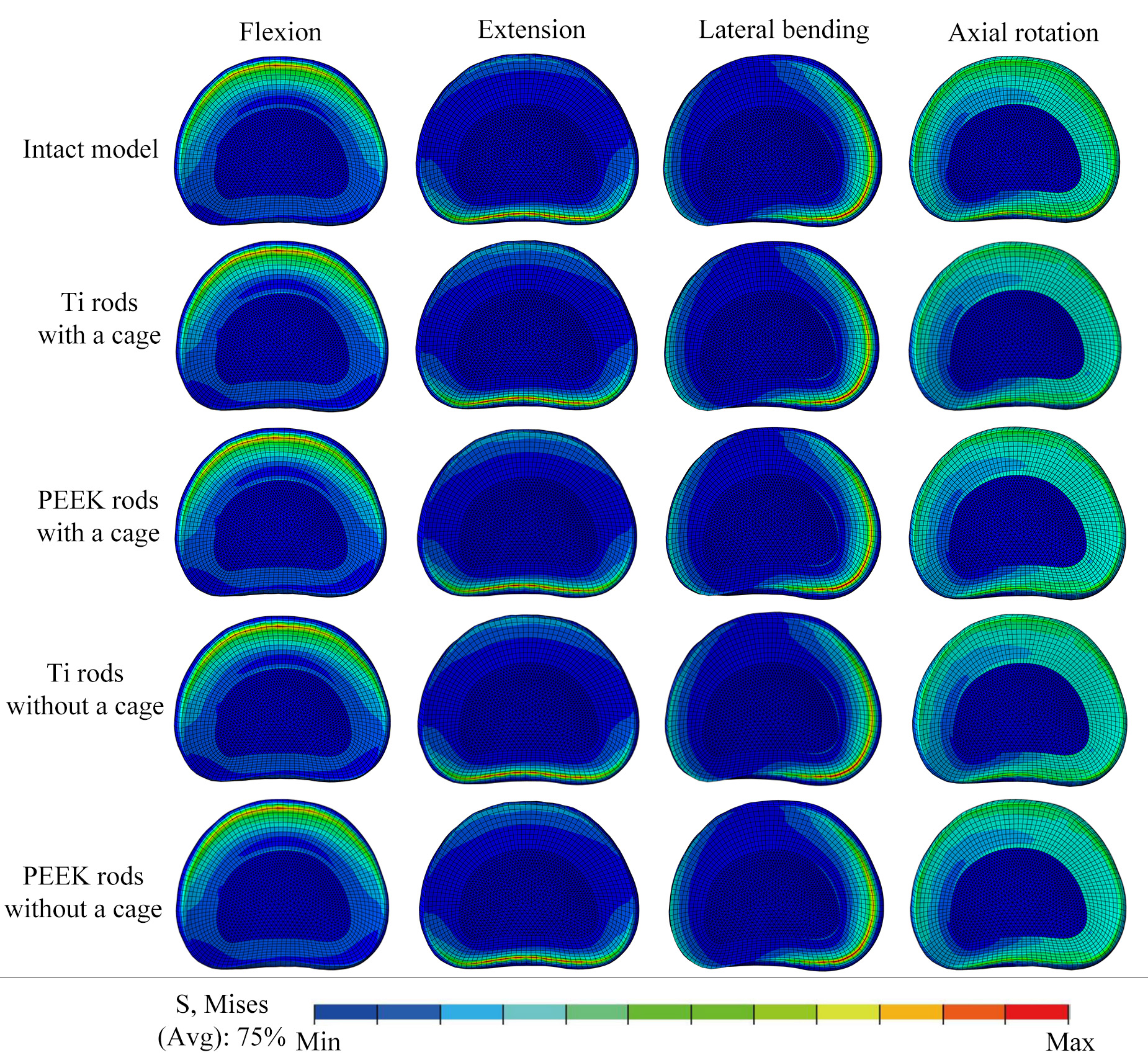

Supplement: Supplementary file 2 — Supplementary Material 2: The nephograms of von Mises stress on the L2/3 disc [file 12893_2024_2462_MOESM2_ESM.jpg]

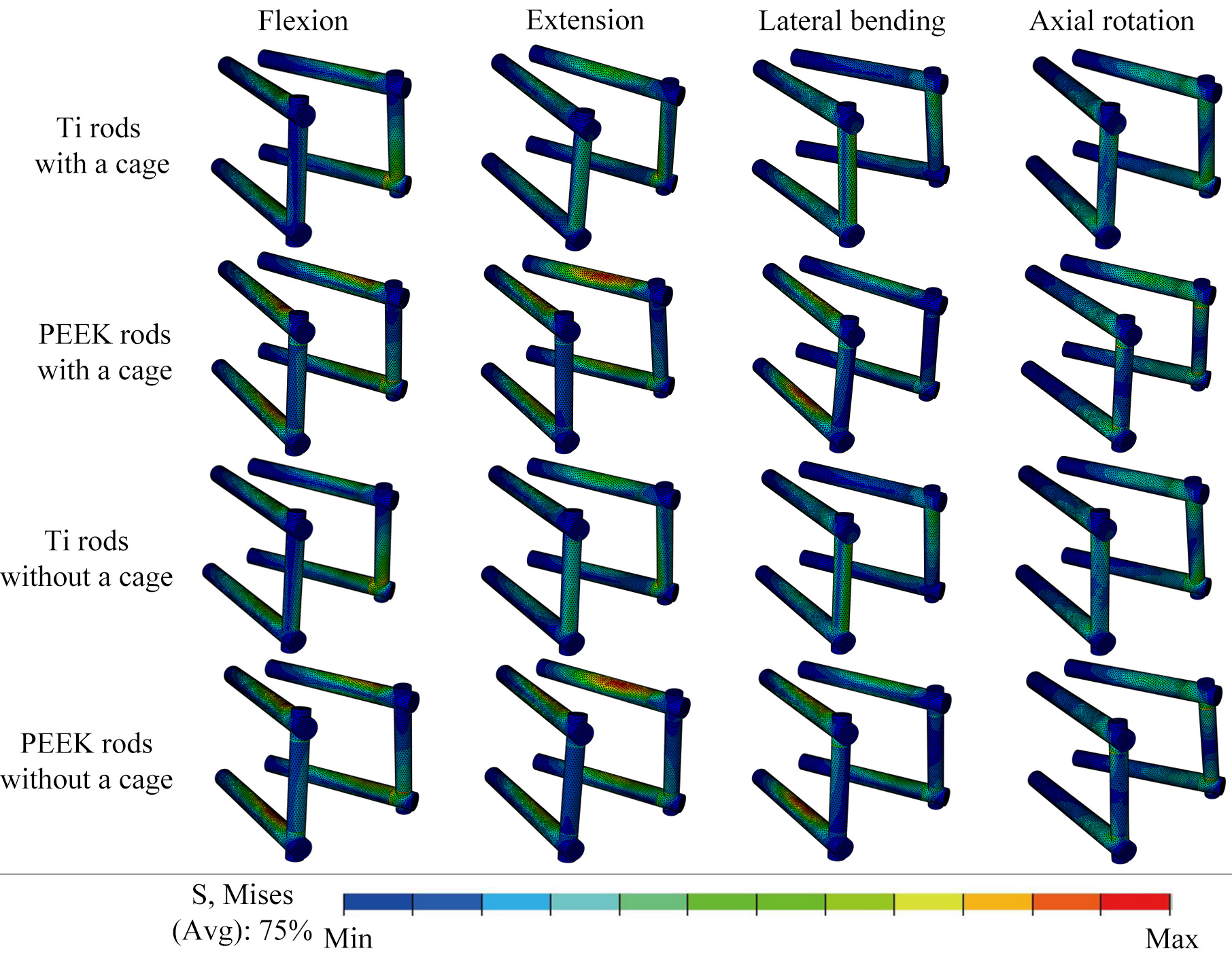

Supplement: Supplementary file 3 — Supplementary Material 3: The nephograms of von Mises stress on the L4/5 disc [file 12893_2024_2462_MOESM3_ESM.jpg]

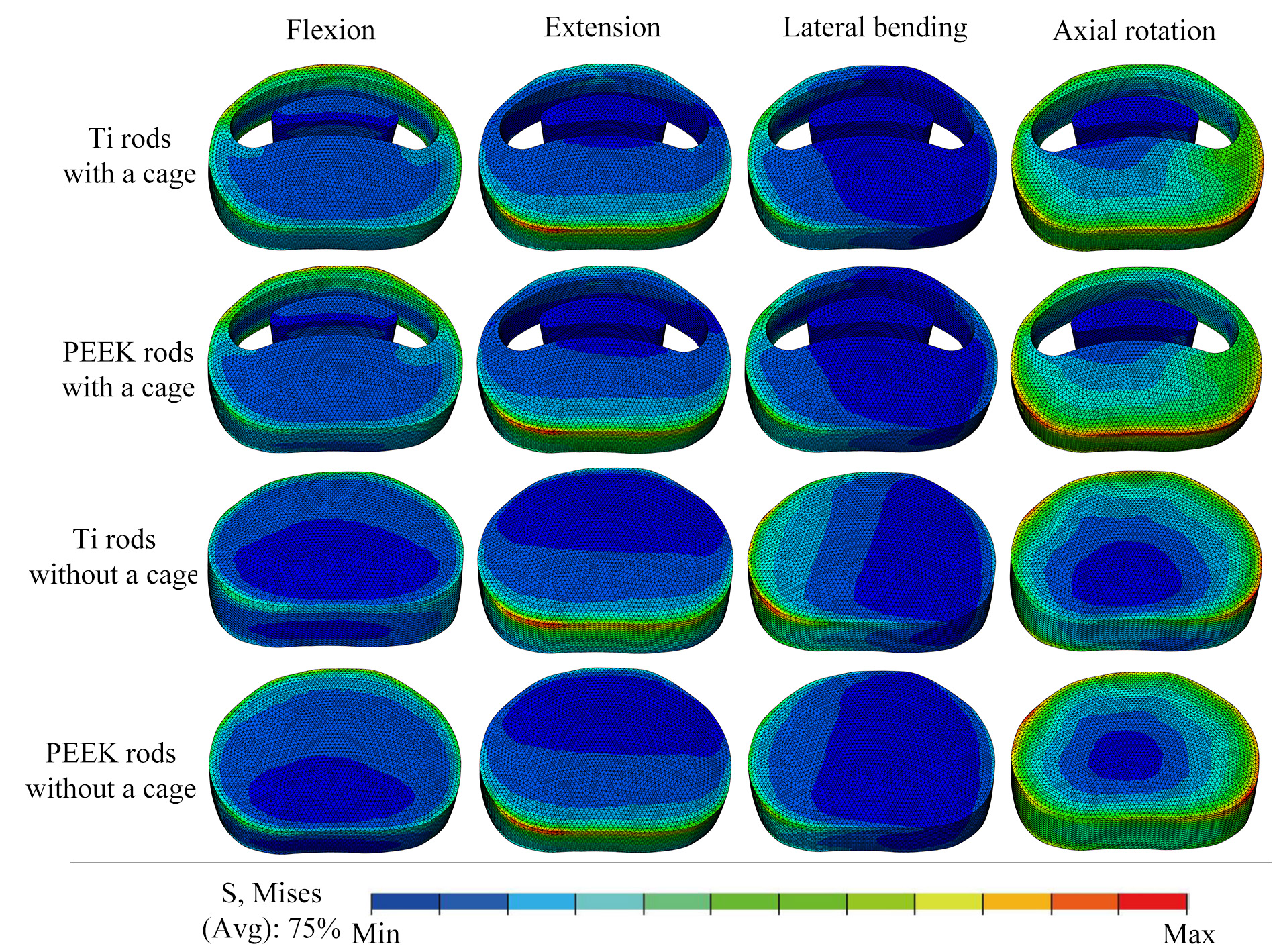

Supplement: Supplementary file 4 — Supplementary Material 4: The nephograms of von Mises stress on the cage [file 12893_2024_2462_MOESM4_ESM.jpg]

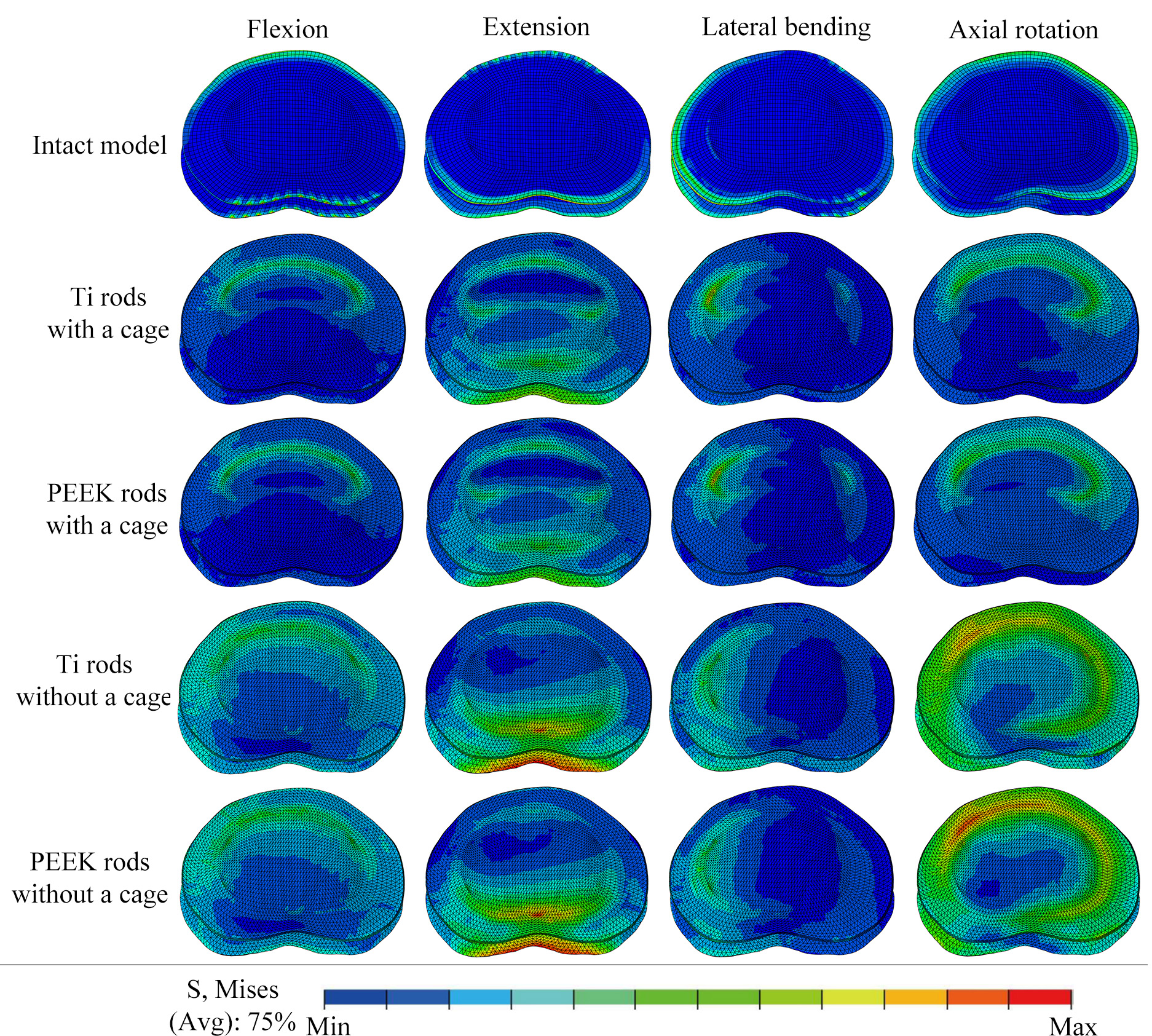

Supplement: Supplementary file 5 — Supplementary Material 5: The nephograms of von Mises stress on the endplates [file 12893_2024_2462_MOESM5_ESM.jpg]

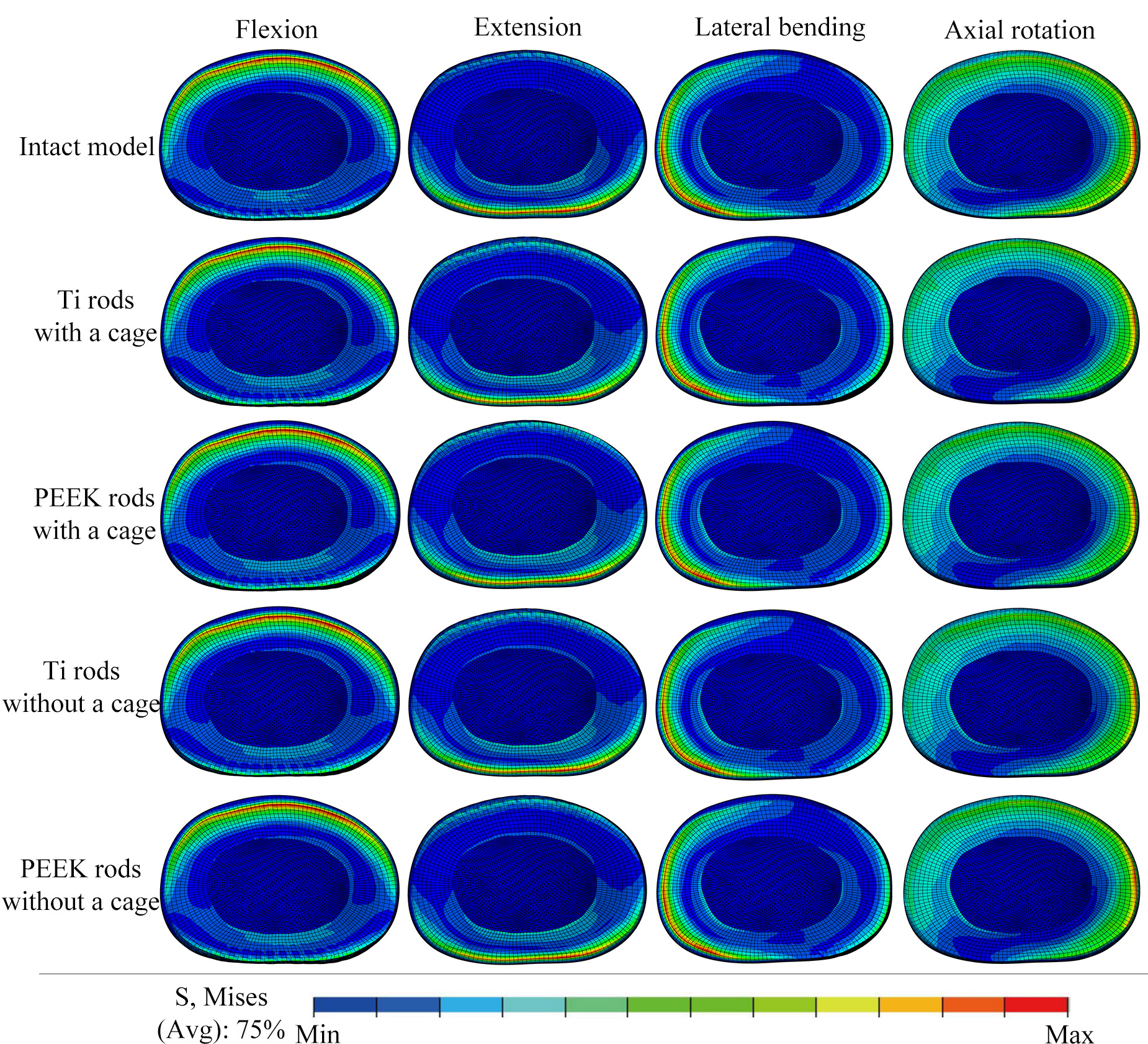

Supplement: Supplementary file 6 — Supplementary Material 6: The nephograms of von Mises stress on the posterior instrumentation [file 12893_2024_2462_MOESM6_ESM.jpg]
